# Kex2 protease converts the endoplasmic reticulum *α*1,2-mannosidase of *Candida albicans* into a soluble cytosolic form

**DOI:** 10.1099/mic.0.2008/019315-0

**Published:** 2008-12

**Authors:** Héctor M. Mora-Montes, Oliver Bader, Everardo López-Romero, Samuel Zinker, Patricia Ponce-Noyola, Bernhard Hube, Neil A. R. Gow, Arturo Flores-Carreón

**Affiliations:** 1 Instituto de Investigación en Biología Experimental, Facultad de Química, Universidad de Guanajuato, Apartado Postal 187, Guanajuato Gto. CP 36000, Mexico; 2 Robert Koch-Institut, FG16, Nordufer 20, D-13353 Berlin, Germany; 3 Departamento de Genética y Biología Molecular, CINVESTAV del IPN, Apartado Postal 14-740, México DF 07000, Mexico; 4 School of Medical Sciences, University of Aberdeen, Aberdeen AB25 2ZD, UK

## Abstract

Cytosolic *α*-mannosidases are glycosyl hydrolases that participate in the catabolism of cytosolic free *N*-oligosaccharides. Two soluble *α*-mannosidases (E-I and E-II) belonging to glycosyl hydrolases family 47 have been described in *Candida albicans*. We demonstrate that addition of pepstatin A during the preparation of cell homogenates enriched *α*-mannosidase E-I at the expense of E-II, indicating that the latter is generated by proteolysis during cell disruption. E-I corresponded to a polypeptide of 52 kDa that was associated with mannosidase activity and was recognized by an anti-*α*1,2-mannosidase antibody. The *N*-mannan core trimming properties of the purified enzyme E-I were consistent with its classification as a family 47 *α*1,2-mannosidase. Differential density-gradient centrifugation of homogenates revealed that *α*1,2-mannosidase E-I was localized to the cytosolic fraction and Golgi-derived vesicles, and that a 65 kDa membrane-bound *α*1,2-mannosidase was present in endoplasmic reticulum and Golgi-derived vesicles. Distribution of *α*-mannosidase activity in a *kex2*Δ null mutant or in wild-type protoplasts treated with monensin demonstrated that the membrane-bound *α*1,2-mannosidase is processed by Kex2 protease into E-I, recognizing an atypical cleavage site of the precursor. Analysis of cytosolic free *N*-oligosaccharides revealed that cytosolic *α*1,2-mannosidase E-I trims free Man_8_GlcNAc_2_ isomer B into Man_7_GlcNAc_2_ isomer B. This is believed to be the first report demonstrating the presence of soluble *α*1,2-mannosidase from the glycosyl hydrolases family 47 in a cytosolic compartment of the cell.

## INTRODUCTION

*N*-Glycosylation is one of the most common post-translational modifications of proteins in eukaryotic cells. The *N*-glycosylation pathway begins in the endoplasmic reticulum (ER), where the dolichol-bound Glc_3_Man_9_GlcNAc_2_ oligosaccharide is assembled, then transferred to nascent proteins and processed by *α*-glycosidases which remove the three glucose residues and one mannose residue to generate the Man_8_GlcNAc_2_ oligosaccharide (Herscovics, 1999a, b). In mammalian cells, *N*-oligosaccharides are further modified to complex glycans by different Golgi glycosyl hydrolases and transferases, whereas in lower eukaryotes such as *Saccharomyces cerevisiae* the only further modifications are by Golgi mannosyl transferases that lead to the biosynthesis of high mannose oligosaccharides (Herscovics, 1999a, b).

*α*-Mannosidases participate in the processing step during *N*-glycan biosynthesis, and in the degradation of *N*-oligosaccharides carried out in the cytosol and acidic compartments such as vacuoles and lysosomes (Daniel *et al.*, 1994; Herscovics, 1999b). These enzymes are also involved in the endoplasmic-reticulum-associated degradation (ERAD) as part of the glycoprotein quality control systems (Helenius & Aebi, 2004). *α*-Mannosidases are grouped into glycosyl hydrolase families 38 (EC 3.2.1.24/EC 3.2.1.114) and 47 (EC 3.2.1.113) (Henrissat & Davis, 1997). Members of family 47 are membrane-bound *α*1,2-mannosidases. Depending on substrate specificity, two types of enzymes can be recognized in the family: those that reside in the ER of yeast and mammalian cells, eliminating a mannose unit from Man_9_GlcNAc_2_ (M_9_) to form Man_8_GlcNAc_2_ isomer B (M_8_B) (Herscovics, 1999a, b), and the Golgi *α*1,2-mannosidases IA, IB and IC, which release the four *α*1,2-linked mannoses from M_9_ to produce Man_5_GlcNAc_2_ (Herscovics, 1999b; Tremblay & Herscovics, 2000).

Golgi *α*-mannosidases II, IIx and III, along with lysosomal, vacuolar acidic and cytosolic/ER neutral *α*-mannosidases, compose family 38. These enzymes are inhibited by swainsonine and can release *α*1,2-, *α*1,3- and *α*1,6-linked mannose residues (Daniel *et al.*, 1994; Herscovics, 1999b).

In lower eukaryotes, cytosolic *α*-mannosidase activity has not been reported; however, in mammals these enzymes have been studied extensively (Dutta & Majumder, 1984; Tulsiani & Touster, 1987; Haeuw *et al.*, 1991; De Gasperi *et al.*, 1992). These neutral cytosolic *α*-mannosidases participate in the hydrolysis of free *N*-oligosaccharides, which are generated by the ERAD of misfolded *N*-glycoproteins and by cleavage of dolichol-linked oligosaccharides (Spiro, 2004; Suzuki *et al.*, 2006). This activity is not sensitive to 1-deoxymannojirimycin (1-DMJ), but is inhibited by swainsonine (Tulsiani & Touster, 1987; Haeuw *et al.*, 1991) and activated by Co^2+^ (Dutta & Majumder, 1984; Haeuw *et al.*, 1991; De Gasperi *et al.*, 1992; Weng & Spiro, 1996). Two cytosolic *α*-mannosidases have been described in rat liver. One trims Man_9_GlcNAc into Man_8_GlcNAc and Man_7_GlcNAc as the ER *α*1,2-mannosidase hydrolyses this oligosaccharide, suggesting that it could be a soluble form of the ER enzyme (Grard *et al.*, 1994). The other is a neutral *α*-mannosidase involved in the catabolism of cytosolic *N*-oligosaccharides released from ER (Grard *et al.*, 1996). Studies of rat liver ER *α*-mannosidase II, which has catalytic and immunological properties similar to the cytosolic *α*-mannosidase, suggest that the cytosolic enzyme may be derived from the ER membrane-bound form (Bischoff & Kornfeld, 1986; Weng & Spiro, 1996). cDNA sequences of rat and mouse ER/cytosolic *α*-mannosidase indicate that they encode soluble proteins, lacking signal sequences, and have homology to the vacuolar *α*-mannosidase from *S. cerevisiae* and other family 38 mannosidases (Bischoff *et al.*, 1990; Costanzi *et al.*, 2006). It has been suggested that this enzyme is synthesized in the cytoplasmic compartment, and then transported to the ER by a mechanism that involves proteolytic processing (Weng & Spiro, 1996; Spiro, 2004). Proteolytic processing also occurs with the vacuolar *α*-mannosidase from *S. cerevisiae* during import from the cytoplasmic compartment to the vacuolar lumen (Yoshihisa & Anraku, 1990).

We have previously purified and characterized two soluble *α*-mannosidases, E-I and E-II, from the human-pathogenic fungus *Candida albicans*. These represent 20 % and 80 % of the total soluble activity, respectively (Vázquez-Reyna *et al.*, 1999; Mora-Montes *et al.*, 2004). Based on the hydrolysis of natural substrates, we proposed that both proteins are *α*1,2-mannosidases belonging to family 47 of glycosyl hydrolases, and that these may be involved in *N*-glycan processing (Mora-Montes *et al.*, 2004, 2006). We have demonstrated that E-I and a membrane-bound *α*-mannosidase activity can be converted into E-II by a proteolytic activity sensitive to pepstatin A (Mora-Montes *et al.*, 2006).

Here, we studied the intracellular localization of *α*1,2-mannosidases of *C. albicans*. A 65 kDa membrane-bound *α*1,2-mannosidase was localized in the ER- and Golgi-derived vesicles, and *α*1,2-mannosidase E-I in the cytosolic and Golgi compartments. However, we found that enzyme E-II is an *in vitro* artefact generated by proteolysis during the preparation of cell extracts. Our data indicate that ER membrane-bound *α*1,2-mannosidase is processed into cytosolic enzyme E-I by Kex2, recognizing an atypical cleavage site. Analysis of the cytosolic fraction of *C. albicans* revealed that E-I is involved in the processing of free M_8_B to Man_7_GlcNAc_2_ isomer B (M_7_B) *N*-oligosaccharide. This study appears to be the first report of an *α*1,2-mannosidase of family 47 in the cytosolic compartment of a lower eukaryote.

## METHODS

### Materials.

M_9_, M_8_B, M_7_B, Man_5_GlcNAc_2_-Asn, 4-methylumbelliferyl-*α*-d-mannopyranoside (MU*α*Man), 4-(2-aminoethyl)benzenesulfonyl fluoride (AEBSF), E64, 1,10-phenanthroline, PMSF, pepstatin A, EGTA, lyticase, sucrose, 3,3′-diaminobenzidine, BSA, monensin, monoclonal anti-rat Golgi 58K protein produced in mouse and anti-mouse IgG-horseradish peroxidase antibody generated in goat were obtained from Sigma. A cocktail of protease inhibitors was obtained from Roche. Anti-yeast carboxypeptidase Y (CPY), anti-rat calnexin and anti-yeast hexokinase I polyclonal sera, all generated in rabbit, were from USBiological. Anti-rabbit IgG-horseradish peroxidase antibody generated in donkey was from Amersham Biosciences. Sepharose CL6B and Sephadex G-25 were from Pharmacia LKB. DEAE Bio-Gel A and Bio-Gel P-6 were purchased from Bio-Rad. All other chemicals were of the highest purity commercially available.

### Organisms and culture conditions.

*Candida albicans* ATCC 26555, CAI4 (Fonzi & Irwin, 1993), SC5314 (Gillum *et al.*, 1984), NGY152 (CAI4-CIp10) (Brand *et al.*, 2004), HMY5 (*mns1*Δ-CIp10), HMY6 (*mns1*Δ-CIp10-*MNS1*) (Mora-Montes *et al.*, 2007) and *kex2*Δ null mutant (Newport *et al.*, 2003), *Candida glabrata* ATCC 2001, *C. glabrata kex2*Δ null mutant (Bader *et al.*, 2001) and *Saccharomyces cerevisiae* X2180-1A (ATCC 26786) were used in this study. They were maintained and propagated in YPD medium [1 % (w/v) yeast extract, 2 % (w/v) mycological peptone, 2 % (w/v) glucose] as described previously (Mora-Montes *et al.*, 2004). *C. albicans* ATCC 26555 was utilized for all experiments unless otherwise indicated.

### Preparation of cell-free extracts and enzyme purification.

Cell-free homogenates were prepared while maintaining all solutions at 4 °C. *C. albicans* yeast cells were collected by low-speed centrifugation, washed twice and resuspended in 50 mM MES/Tris buffer, pH 6.0 (buffer A), with or without protease inhibitors and were broken in a Braun MSK cell homogenizer using 0.45 mm diameter glass beads. The homogenate was centrifuged at 1000 ***g*** for 10 min and the supernatant was collected and further centrifuged at 105 000 ***g*** for 1 h (ultracentrifugation). The high-speed supernatant was collected, freeze-dried and kept at –20 °C until use. In some experiments, the pellet, consisting of a mixed-membrane fraction, was resuspended in buffer A and used to assay *α*-mannosidase activity.

For enzyme E-I purification, the high-speed supernatant obtained in the presence of 1 μM pepstatin A was purified by size-exclusion, anionic-interchange and concanavalin A-Sepharose affinity chromatography as described previously (Mora-Montes *et al.*, 2006). To purify the membrane-bound *α*-mannosidase from *C. albicans*, *C. glabrata* and *S. cerevisiae*, the mixed-membrane fraction was incubated for 1 h at 48 °C, followed by incubation at −20 °C for 30 min. This procedure solubilized about 50 % of the non-enzymic protein present in the mixed-membrane fraction, without significantly affecting the total *α*-mannosidase activity remaining in the pellet. Then, the preparation was ultracentrifuged, and the pellet was recovered, resuspended in 1 ml of buffer A with 3 mM EDTA, incubated 1 h at room temperature and ultracentrifuged as indicated. The pellet was resuspended in 1 ml buffer A containing 0.5 M NaCl, incubated for 1 h at room temperature and ultracentrifuged. Finally, the pellet was resuspended in 1 ml buffer A added with Triton X-100 in a protein : detergent ratio of 1 : 1, and was incubated for 1 h at room temperature and ultracentrifuged. The pellet was then resuspended in 1 ml buffer B (50 mM Tris, 0.2 M KCl, 1 mM CaCl_2_, 0.01 % Triton X-100, pH 7.2) and kept at −20 °C until use.

### *α*-Mannosidase assay.

Enzyme activity was measured using either the fluorogenic substrate MU*α*Man or the natural oligosaccharides M_9_, M_8_B or Man_5_GlcNac_2_-Asn, essentially as described previously (Mora-Montes *et al.*, 2004).

### Protein determination.

Protein was measured by absorbance at 280 nm and by the method of Bradford (1976) using BSA as a standard.

### Electrophoresis.

SDS-PAGE was carried out using 10 % gels following standard protocols (Laemmli, 1970), and proteins were stained with Coomassie blue (Merril, 1990). Zymogram analysis in SDS-PAGE, using the fluorogenic substrate MU*α*Man, was performed for the enzyme detection as described previously (Mora-Montes *et al.*, 2004).

### Protoplast generation and differential centrifugation of homogenates.

Protoplasts were obtained following the protocol described by Ramírez *et al.* (1989). Briefly, yeast cells were collected by low-speed centrifugation, resuspended at an OD_600_ of 2–3 in buffer C (50 mM Tris/HCl buffer, pH 7.5, 1 M sorbitol, 0.8 M KCl and 10 mM MgSO_4_), lyticase (0.25 mg ml^−1^) and 15 mM *β*-mercaptoethanol. After incubation at 37 °C for 30 min, nearly 100 % of the cells had been converted into protoplasts. These were washed twice with buffer C and resuspended in 10 mM phosphate buffer, pH 6.0, containing 3.0 mM MgCl_2_ (buffer D). Samples of protoplasts were lysed with a Potter–Elvehjem and pestle (8–10 strokes) and the lysates were centrifuged at 1000 ***g*** for 10 min. The resulting supernatants were collected and 4 ml aliquots were loaded onto the top of a 35 ml, continuous 10–65 % (w/w) sucrose density gradient prepared with buffer D and centrifuged at 232 000 *g* for 4 h at 4 °C using a VTi 50 rotor (Beckman Coulter). Gradients were fractionated from the top and 1 ml fractions were collected. Alternatively, the protoplasts were incubated with 10 μM monensin for 1 h at 37 °C and lysed as described above.

### Determination of free *N*-oligosaccharides in the cytosol.

Homogenates of protoplasts of *C. albicans*, *C. glabrata* or *S. cerevisiae*, treated or untreated with monensin, were fractionated in a continuous 10–65 % (w/w) sucrose density gradient as indicated above. The soluble fraction (typically fractions 1–4) was saved, freeze-dried, resuspended in 150 μl deionized water and applied onto a column (0.3×105 cm) of Bio-Gel P-6. Fractions of 120 μl were collected and those enriched with the oligosaccharides were pooled, and analysed by high-performance anion-exchange chromatography as described previously (Mora-Montes *et al.*, 2004).

### Antibodies.

The anti-*α*1,2-mannosidase antibody was generated from recombinant protein as follows. The open reading frame of the *C. albicans MNS1* gene (GenBank/EBI accession AY167027) was cloned into the bacterial expression vector pET100/D-TOPO (Invitrogen), overexpressed in *Escherichia coli* and the recombinant protein was purified. Antibodies were raised in a male New Zealand White rabbit after intramuscular injection of 150 μg protein emulsified with complete Freund's adjuvant (day 0). Booster injections were given (150 μg protein emulsified with incomplete Freund's adjuvant) on days 15, 30, 45 and 60 and the animal was bled on day 75.

Anti-calnexin, anti-*α*1,2-mannosidase, rabbit pre-immune serum, anti-CPY, anti-hexokinase I and anti-Golgi primary antibodies were diluted 1 : 1000, 1 : 3000, 1 : 3000, 1 : 5000, 1 : 5000 and 1 : 5000, respectively, in PBS. The secondary, anti-mouse IgG-horseradish peroxidase and anti-rabbit IgG-horseradish peroxidase antibodies were diluted 1 : 2000 in PBS.

### Immunoblots.

Purified protein or samples (10 μg) of each *α*-mannosidase peak, separated after density-gradient fractionation of protoplast homogenates, were subjected to SDS-PAGE (10 %) and electrophoretically transferred to Hybond-C extra nitrocellulose membranes following standard protocols. The membranes were stained with Ponceau S Red to assess the efficiency of transfer. The membranes were blocked with 1 % BSA in PBS for 2 h, washed three times with 0.05 % Tween 20 in PBS for 10 min, and the primary antibody was added and incubated for 2 h at room temperature. Membranes were then washed three times with 0.05 % Tween 20 in PBS for 10 min, incubated for 2 h with the secondary antibody, and washed twice with 0.05 % Tween 20 in PBS for 10 min and once with PBS for 10 min. Bands were revealed with 3,3′-diaminobenzidine (0.5 mg ml^−1^) and 3 % hydrogen peroxide in PBS. Reactions were stopped with deionized water.

### Preparation of Kex2 enzymes.

*S. cerevisiae* and *C. glabrata* secreted, soluble Kex2 proteins were produced in a commercial *Pichia pastoris* expression system (Invitrogen). The strain expressing *S. cerevisiae* Kex2 was a kind gift of G. Boileau (Lesage *et al.*, 2001). The *C. glabrata* enzyme was produced in a similar manner. The *C. albicans* secreted, soluble form of the enzyme was expressed from the *ACT1* promoter in *C. albicans* strain CAI4. All enzyme preparations were purified by a combination of size-exclusion and anion-exchange chromatography and tested with specific substrates and inhibitors to ascertain their specificity (O. Bader & B. Hube, unpublished data).

### Proteolytic cleavage of membrane-bound *α*-mannosidase.

Twenty micrograms of protein from peak 2 and peak 3, obtained after treatment with 10 μM monensin (see above), was resuspended in 50 μl buffer D added with 0.5 % Triton X-100, with or without protease inhibitors, and incubated for 1 h at 37 °C with gentle shaking. The reactions were analysed by immunoblotting using the anti-*α*1,2-mannosidase antibody. For the processing using recombinant Kex2, 20 μg of the partially purified membrane-bound *α*-mannosidase and 3 μg of recombinant Kex2 from *S. cerevisiae*, *C. albicans* or *C. glabrata* were resuspended in buffer B in a final volume of 20 μl, and incubated for 1 h at 37 °C with gentle shaking. Then, the reactions were applied to 4–12 % NuPAGE Bistris gels (Invitrogen) and analytic zymograms were carried out (see above).

### N-terminal sequencing.

The purified *α*-mannosidase E-I and the 52 kDa *α*-mannosidase generated by Kex2 were subjected to double-dimension electrophoresis using isoelectrofocusing ZOOM pH 4–7 strips and 4–12 % NuPAGE Bistris ZOOM gels (Invitrogen). The gels were washed three times with buffer A added with 1 % Triton X-100 for 20 min at room temperature, and *α*-mannosidase activity was detected after incubation with buffer A added with 40 μM MU*α*Man for 1 h at 37 °C. The proteins were transferred to a PVDF membrane and N-terminal sequencing was carried out by BioSynthesis.

## RESULTS

### Effect of protease inhibitors on intracellular distribution of *α*-mannosidase activity

Yeast cells of *C. albicans* were broken in the presence of protease inhibitors and the resulting homogenates were subjected to ultracentrifugation. *α*-Mannosidase activity was measured in the high-speed supernatant and in the mixed-membrane fraction. The results, summarized in Table 1, indicate that of the inhibitors tested, only pepstatin A affected the subcellular distribution of the enzyme. In the absence of pepstatin A, *α*-mannosidase was recovered mostly in the supernatant (78–82 %) while only 18–22 % remained associated with the membrane fraction. However, in homogenates prepared in the presence of pepstatin A, total activity was distributed in approximately equal proportions in the soluble (44 %) and membrane (56 %) fractions.

**Fig. 1. f1:**
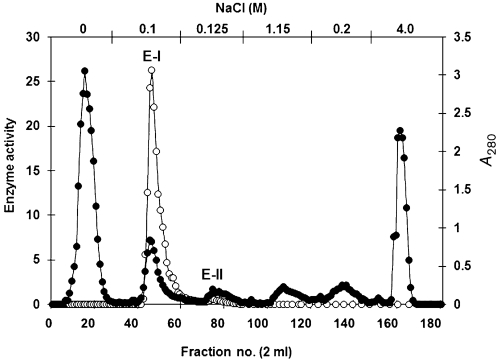
Separation of *α*-mannosidase E-I by ion-exchange chromatography. High-speed supernatant from pepstatin A-treated homogenates was separated by gel filtration in Sepharose CL6B and the enzyme fraction obtained was applied on a column (2.8×7.3 cm) of DEAE Bio-Gel A equilibrated with buffer A. After washing with the same buffer, bound proteins were eluted with a discontinuous gradient of 0–4.0 M NaCl in buffer A. Fractions were collected and used to monitor elution of protein (filled symbols) and enzyme activity [nmol MU min^−1^ (mg protein)^−1^] using MU*α*Man as substrate (open symbols).

The ratio of the soluble E-I and E-II enzymes could be determined after separation of these two soluble activities by ion-exchange chromatography. In the presence of pepstatin A, only a single peak of *α*-mannosidase representing E-I was eluted by 0.1 M NaCl from a DEAE Bio-Gel A column. After eluting with 0.125 M NaCl, a second barely detectable peak corresponding to *α*-mannosidase E-II was obtained (Fig. 1).

**Fig. 2. f2:**
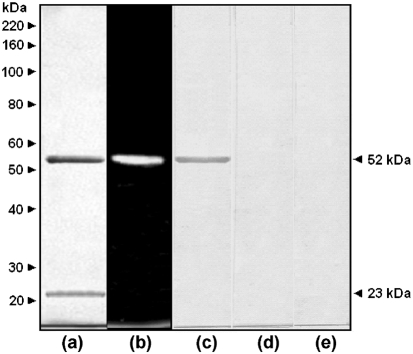
Analytical zymogram and immunodetection of purified *α*-mannosidase. The purified enzyme (10 μg) was analysed by SDS-PAGE (10 %) either after heat denaturation (a) or without heating (b) and separated by electrophoresis. Lane (a) was stained with Coomassie blue and lane (b) was incubated with MU*α*Man, as described in the text, to reveal the *α*-mannosidase activity. After separation by SDS-PAGE, proteins were electrotransferred to nitrocellulose membranes and immunodetected with anti-*α*1,2-mannosidase antibody (c), rabbit pre-immune serum (d) or anti-rabbit IgG-horseradish peroxidase antibody (e).

The enzyme E-I was purified from pepstatin A-treated extracts using a combination of size-exclusion, ion-exchange and affinity chromatography as described previously (Mora-Montes *et al.*, 2006). Following this protocol, the enzyme E-I was purified 230-fold with a recovery of 34 % of the starting material (Table 2). Analytical electrophoresis of the purified sample revealed the presence of two polypeptides, of 23 and 52 kDa (Fig. 2a[Fig f2]). Only the latter was active on the fluorogenic substrate MU*α*Man as revealed by zymogram analysis (Fig. 2b[Fig f2]), and was recognized by the anti-*α*1,2-mannosidase antibody (Fig. 2c[Fig f2]). No immunorecognition was observed in samples containing pre-immune serum or lacking the primary antibody (Fig. 2d, e[Fig f2]).

The processing of natural substrates such as M_9_ oligosaccharide by the purified enzyme was similar to that described previously (Mora-Montes *et al.*, 2006). This resulted in the production of M_8_B and mannose as the sole products of hydrolysis after 12 h of incubation, and M_7_B and Man_6_GlcNAc_2_ after 24 h of incubation (data not shown). As was described for *α*-mannosidases E-I and E-II (Mora-Montes *et al.*, 2004, 2006), the purified enzyme did not hydrolyse Man_5_GlcNAc_2_-Asn oligosaccharide (data not shown) and was more sensitive to 1-DMJ than to swainsonine, with IC_50_ values of 0.22 mM and 0.54 mM, respectively.

### Subcellular localization of *α*-mannosidase E-I

**Fig. 3. f3:**
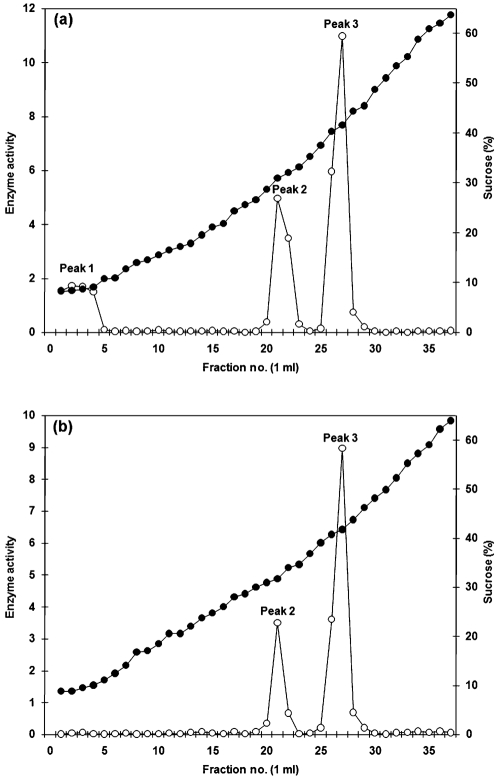
Distribution of *α*-mannosidase activity after density-gradient centrifugation of homogenates from *C. albicans* and *S. cerevisiae*. Protoplasts from *C. albicans* (a) or *S. cerevisiae* (b) were prepared and homogenized as described in Methods. A 4 ml sample of each homogenate was loaded onto the top of a 35 ml, continuous 10–65 % (w/w) sucrose density gradient and centrifuged at 232 000 *g* at 4 °C for 4 h in a VTi 50 rotor. The gradient was fractionated from the top and 1 ml fractions were collected. These were used to monitor enzyme activity [nmol MU min^−1^ (mg protein)^−1^] using MU*α*Man as substrate (open symbols) and the sucrose concentration (filled symbols). Fraction 1 corresponds to the top of the gradient.

To investigate the subcellular localization of enzyme E-I, gently disrupted protoplasts of *C. albicans* were subjected to centrifugation in a continuous, 10–65 % sucrose density gradient (see Methods). *α*-Mannosidase activity was distributed in three peaks associated with fractions 1–4 (peak 1), 21–22 (peak 2) and 26–27 (peak 3) with corresponding densities of 1.033±0.002 g cm^−3^, 1.130±0.007 g cm^−3^ and 1.183±0.004 g cm^−3^ and representing 20 %, 27 % and 53 % of total recovered activity, respectively (Fig. 3a[Fig f3]). Centrifugation of a homogenate from protoplasts of *S. cerevisiae* under the same conditions resulted in separation of two peaks of *α*-mannosidase activity associated with fractions 21–22 (peak 2) and 26–27 (peak 3) with corresponding densities of 1.137±0.006 g cm^−3^ and 1.184±0.006 g cm^−3^, respectively. Peaks 2 and 3 represented 26 % and 74 % of total recovered activity, respectively (Fig. 3b[Fig f3]).

**Fig. 4. f4:**
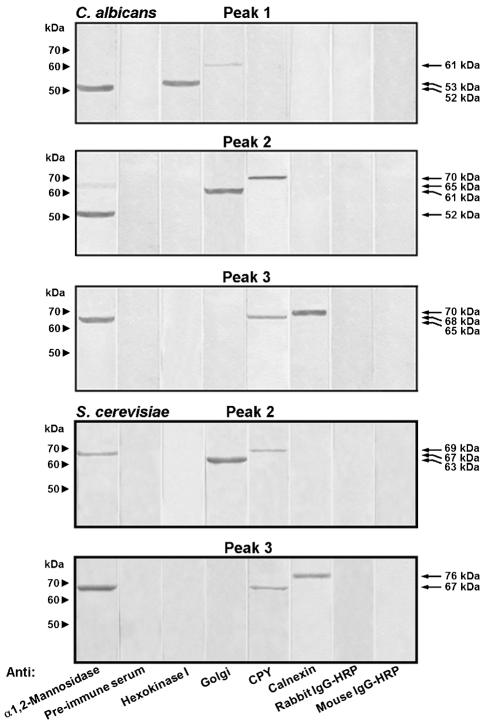
Immunodetection of *α*1,2-mannosidase and some organelle markers in fractions separated as indicated in Fig. 3. Protein samples (10 μg) of *α*-mannosidase peaks from *C. albicans* and *S. cerevisiae* were separated by SDS-PAGE, electrotransferred to nitrocellulose membranes and immunodetected with anti-*α*1,2-mannosidase and organelle markers as described in Methods. Controls used rabbit pre-immune serum (Pre-immune serum), anti-rabbit IgG-horseradish peroxidase (Rabbit IgG-HRP) or anti-mouse IgG-horseradish peroxidase (Mouse IgG-HRP).

Immunoblot assays using antibodies against *α*1,2-mannosidase and several organelle-marker proteins were carried out to determine the presence of *α*-mannosidase E-I in the peaks observed in Fig. 3(a). The results, illustrated in Fig. 4, indicate that the anti-*α*1,2-mannosidase antibody detected a polypeptide of 52 kDa in peaks 1 and 2, which corresponds to the molecular mass of *α*-mannosidase E-I (see above). The same antibody detected trace amounts of a 65 kDa protein in peak 2 which was enriched in peak 3. Hexokinase I, a cytosolic marker (Huh *et al.*, 2003), was only detected in peak 1, while a Golgi marker was barely detected in peak 1 but was abundant in peak 2. CPY, a marker of acid vacuoles (Bryant & Stevens, 1998), was present in peaks 2 and 3, and calnexin, an ER marker (Parlati *et al.*, 1995), was only detected in peak 3. Mock controls containing pre-immune serum or lacking the primary antibodies run in parallel gave no detectable signals. Therefore E-I was enriched in cytosolic and Golgi compartments. The anti-*α*1,2-mannosidase antibodies detected a 67 kDa polypeptide in both *α*-mannosidase peaks separated on sucrose density gradients of extracts from protoplasts of *S. cerevisiae* (Fig. 4). Results with organelle markers were similar to those obtained with *α*-mannosidase peaks from the *C. albicans* preparation. These results confirmed that *S. cerevisiae* had only one *α*1,2-mannosidase, whereas *C. albicans* had two isoforms of *α*1,2-mannosidase.

**Fig. 5. f5:**
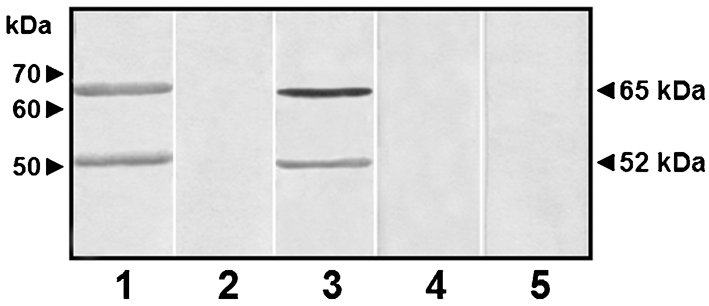
Immunodetection of *α*1,2-mannosidase in cell homogenates from *mns1*Δ null mutant, wild-type strain and reintegrant control. Cell homogenates from strains NGY152 (wild-type strain, lanes 1 and 4), HMY5 (*mns1*Δ null mutant, lane 2), and HMY6 (reintegrant control, lanes 3 and 5) were prepared as described in Methods, and 25 μg protein samples were separated by SDS-PAGE, electrotransferred to nitrocellulose membranes and immunodetected with anti-*α*1,2-mannosidase (lanes 1–3) or pre-immune (lanes 4 and 5) sera.

An anti-*α*1,2-mannosidase antibody was raised against the *MNS1* product (see Methods), which is a 65 kDa *α*1,2-mannosidase that is predicted to be localized in the ER (Mora-Montes *et al.*, 2007). Western analysis showed that Mns1 and *α*1,2-mannosidase E-I were both detected by this antibody. In order to demonstrate that both *α*1,2-mannosidase isoforms are encoded by *MNS1*, immunoblot assays using anti-*α*1,2-mannosidase antibodies and cell homogenate from a *mns1*Δ null mutant were carried out. Neither protein was detected in cell homogenates from *mns1*Δ null mutant (Fig. 5, lane 2), whilst two protein bands with molecular masses of 65 and 52 kDa were recognized by the antibodies in the cell homogenates from the wild-type strain (Fig. 5, lane 1) and the reintegrant control (Fig. 5, lane 3). Control assays using pre-immune serum did not give any detectable signal (Fig. 5, lanes 4 and 5). Additionally, the *mns1*Δ null mutant did not show any hydrolytic activity towards the natural substrate M_9_ (data not shown). Therefore, *MNS1* encodes both 65 kDa and 52 kDa, *α*1,2-mannosidase isoforms.

### Effect of monensin on the localization of soluble *α*1,2-mannosidase

**Fig. 6. f6:**
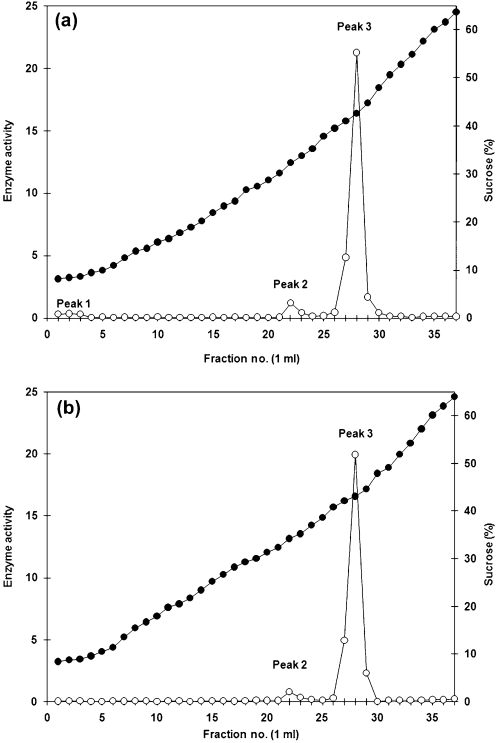
Distribution of *α*-mannosidase activity of monensin-treated homogenates from *C. albicans* and *S. cerevisiae*. Details as for Fig. 3, but the protoplasts from *C. albicans* (a) or *S. cerevisiae* (b) were incubated with 10 μM monensin for 1 h at 37 °C before the homogenization and separation in a continuous 10–65 % (w/w) sucrose density gradient. *α*-Mannosidase activity [nmol MU min^−1^ (mg protein)^−1^] using MU*α*Man as substrate (open symbols) and the sucrose concentration (filled symbols) are indicated.

The results in Fig. 4 indicate that *α*1,2-mannosidase E-I is associated with the cytosolic and Golgi markers, whereas a 65 kDa *α*1,2-mannosidase was enriched in the ER and to a lesser extent in Golgi fractions. To determine whether E-I was generated from the 65 kDa protein, the effect of monensin on *α*1,2-mannosidase localization was analysed. Monensin, a monovalent ion-selective ionophore, blocks the transport of vesicles from the medial to the trans cisternae of the Golgi complex (Rosa *et al.*, 1992), leading to an accumulation of proteins in the ER. Protoplasts of *C. albicans* were incubated with 10 μM monensin (see Methods) before their disruption and fractionation in a continuous 10–65 % sucrose density gradient. Under these conditions, the *α*-mannosidase activity was not affected (data not shown) and was again distributed in three peaks with corresponding densities of 1.032±0.003 g cm^−3^ for peak 1 (fractions 1–3), 1.139±0.007 g cm^−3^ for peak 2 (fractions 22–23), and 1.184±0.002 g cm^−3^ for peak 3 (fractions 27–28). The *α*-mannosidase activity associated with peaks 1 and 2 was significantly decreased to 3 % and 6 %, respectively, and the rest of the recovered *α*-mannosidase activity (91 %) was associated with peak 3 (Fig. 6a[Fig f6]). Anti-*α*1,2-mannosidase antibodies barely detected *α*-mannosidase E-I in peaks 1 and 2, whereas the 65 kDa *α*1,2-mannosidase was abundant in peak 3 (not shown). When protoplasts of *S. cerevisiae* were treated in the same way, the *α*-mannosidase activity was distributed in two peaks associated with fractions 22–23 (peak 2) and 27–28 (peak 3) with corresponding densities of 1.140±0.003 g cm^−3^ and 1.186±0.005 g cm^−3^, respectively. As for the monensin-treated protoplasts of *C. albicans*, there was a redistribution of the *α*-mannosidase activity associated with peaks 2 and 3, representing 4 % and 96 % of total recovered activity, respectively (Fig. 6b[Fig f6]).

**Fig. 7. f7:**
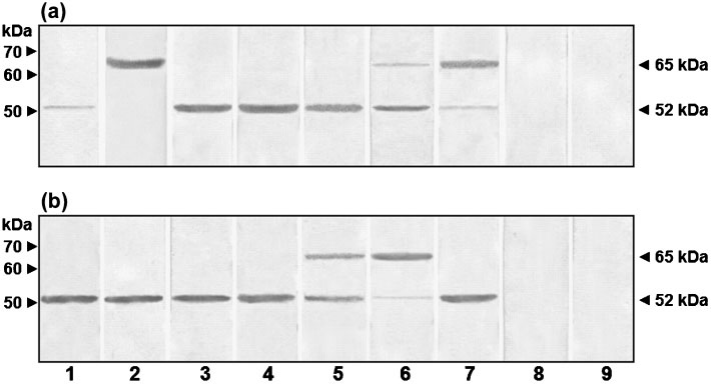
Cleavage of 65 kDa *α*1,2-mannosidase into *α*1,2-mannosidase E-I by a Golgi-associated protease activity. Aliquots containing 10 μg protein were separated by SDS-PAGE, electrotransferred to nitrocellulose membranes and immunodetected with anti-*α*1,2-mannosidase (a, b, lanes 1–7), rabbit preimmune (a, b, lane 8) or anti-rabbit IgG-horseradish peroxidase (a, b, lane 9) sera. Lanes 1 and 2 in (a) correspond to samples from *α*-mannosidase peaks 2 and 3, respectively, separated after treatment with monensin. The samples were incubated with 0.5 % Triton X-100 for 1 h at 37 °C before the electrophoresis. Lane 3 in (a) and lane 1 in (b) contain protein samples from peaks 2 and 3 pooled and incubated as described above. The incubations of both peaks were also carried out in the presence of 0.5, 1.0, 5.0 and 10 mM PMSF (a, lanes 4–7); 10 μM pepstatin A (b, lane 2); 1 mM E-64 (b, lane 3); 20 mM 1,10-phenanthroline (b, lane 4); 0.5 and 1.0 mM EGTA (b, lanes 5 and 6); 1.0 mM EGTA plus 3 mM CaCl_2_ (b, lane 7).

These results suggest that *α*-mannosidase E-I is generated from the 65 kDa *α*1,2-mannosidase by proteolysis in the Golgi compartment. Supporting this, we found that proteolytic processing of the 65 kDa *α*1,2-mannosidase to the 52 kDa form occurred if we used ER vesicles from monensin-treated cells as a source of the *α*1,2-mannosidase, and Golgi vesicles as a source of the processing protease (Fig. 7). The generation of 52 kDa *α*1,2-mannosidase E-I depended on addition of Triton X-100 to the incubations to solubilize the proteins from the vesicles (data not shown).

In order to establish the nature of the proteolytic activity, similar experiments were carried out in the presence of protease inhibitors (Fig. 7). Addition of PMSF at 0.5 mM (panel a, lane 4) or 1.0 mM (panel a, lane 5) was unable to prevent the generation of *α*1,2-mannosidase E-I; however, 5.0 mM PMSF partially inhibited the proteolytic processing of 65 kDa protein (panel a, lane 6). This processing was almost completely inhibited when the concentration of PMSF was increased to 10 mM (panel a, lane 7). High concentrations of pepstatin A (10 μM), *trans*-epoxysuccinyl-l-leucyl-amido(4-guanidino)butane (E-64) (1 mM) or 1,10-phenanthroline (20 mM) did not affect the conversion of 65 kDa *α*1,2-mannosidase into *α*-mannosidase E-I (panel b, lanes 2–4). Addition of 0.5 mM EGTA partially inhibited the proteolytic processing (panel b, lane 5), and almost complete inhibition occurred using 1.0 mM EGTA (panel b, lane 6). The EGTA effect was prevented by the addition of 3 mM CaCl_2_ (panel b, lane 7). Mock controls containing preimmune serum (panels a and b, lane 8) or lacking the primary antibodies (panels a and b, lane 9) run in parallel gave no detectable signals. Therefore a Ca^2+^-dependent proteolysis is required for the processing of ER *α*1,2-mannosidase activity into E-I.

### Conversion of 65 kDa *α*1,2-mannosidase into enzyme E-I by Kex2

**Fig. 8. f8:**
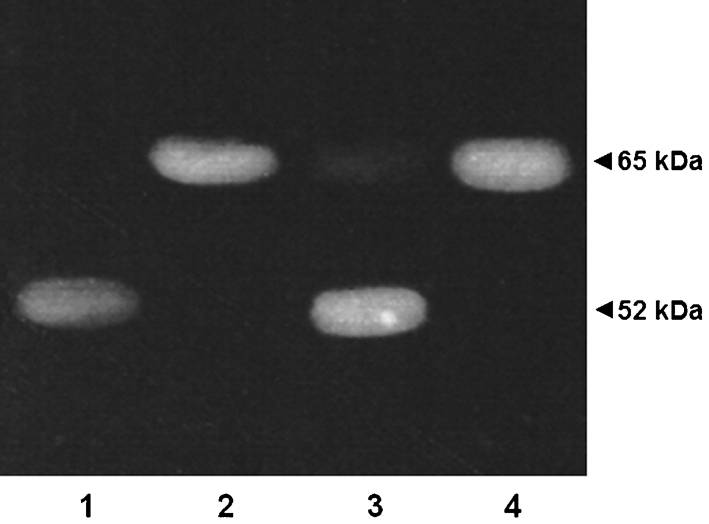
Proteolytic processing of 65 kDa *α*1,2-mannosidase into *α*1,2-mannosidase E-I by recombinant Kex2. Samples (20 μg) of the purified 65 kDa *α*1,2-mannosidase were incubated with 3 μg recombinant Kex2 from *S. cerevisiae* for 1 h at 37 °C in the absence (lane 3) or presence (lane 4) of 1 mM EGTA. The reactions were analysed by analytical zymograms with MU*α*Man as described in Methods. Control reactions containing only purified *α*1,2-mannosidase E-I (lane 1) or 65 kDa *α*1,2-mannosidase (lane 2) were run in parallel.

The Golgi protease activity that processed the 65 kDa *α*1,2-mannosidase into *α*1,2-mannosidase E-I was sensitive to high concentrations of PMSF and EGTA. This inhibition profile is similar to that observed for the Ca^2+^-dependent protease Kex2 (Fuller *et al.*, 1988, 1989). Hence, we compared the *α*-mannosidase activity distribution in a *C. albicans kex2*Δ null mutant and in the wild-type strain CAI4. For strain CAI4 [total *α*-mannosidase activity 94.6±5.3 nmol 4-methylumbelliferone (MU) min^−1^ (mg total protein)^−1^], 46 % of the total activity was present in a soluble form as previously demonstrated (Mora-Montes *et al.*, 2006); however in the *kex2*Δ null mutant [total *α*-mannosidase activity 90.8±4.8 nmol MU min^−1^ (mg total protein)^−1^] more than 99 % of the *α*-mannosidase activity was associated with the mixed-membrane fraction. When wild-type or *kex2*Δ null mutant cells were broken in the presence of 1 mM EGTA, the *α*-mannosidase distribution was similar to that of untreated cells. These results suggest that Kex2 participates in the generation of *α*-mannosidase E-I. To determine whether Kex2 was able to process the 65 kDa *α*1,2-mannosidase into E-I, incubations of purified 65 kDa *α*1,2-mannosidase from mixed-membrane fraction and recombinant Kex2 from *S. cerevisiae* were carried out, and analysed in zymograms using the fluorogenic substrate MU*α*Man (see Methods). The results, illustrated in Fig. 8, indicate that Kex2 processed the membrane-bound 65 kDa *α*1,2-mannosidase into *α*-mannosidase E-I (lane 3), and this proteolytic cleavage was sensitive to EGTA (lane 4). The 65 kDa *α*1,2-mannosidase and E-I were run as controls (lanes 1 and 2). Similar results were obtained when we analysed the products with the anti-*α*1,2-mannosidase antibodies, or when we performed the reactions using ER membrane-bound *α*1,2-mannosidase from *C. glabrata* or recombinant Kex2 from *C. albicans* or *C. glabrata* (data not shown). However, recombinant Kex2 failed to hydrolyse the ER membrane-bound *α*1,2-mannosidase from *S. cerevisiae* (data not shown).

*α*-Mannosidase of 52 kDa generated by Kex2 processing (Fig. 8, lane 3) and the previously purified *α*-mannosidase E-I (Fig. 2) were subjected to N-terminal sequencing, and for both proteins the identical amino acid sequence Asp-Trp-Ile-Lys-Asn-Asp-Leu-Asp-Tyr-Thr-Phe-Asp-Tyr-Asn-Val-Asn-Thr-Phe-Glu was obtained. This sequence was localized at position 103–121 within the sequence for the *MNS1* product. Therefore, the same Kex2-dependent proteolytic product was apparently generated *in vitro* and *in vivo*.

### Effect of monensin on free *N*-oligosaccharides present in the cytosol

To obtain further insights into the role of *α*1,2-mannosidase E-I in the cytosolic compartment, the soluble fraction of protoplasts of *C. albicans* was recovered after fractionation in a continuous, 10–65 % sucrose density gradient (typically fractions 1–4) and was analysed by high-performance anion-exchange chromatography (see Methods). M_8_B and M_7_B were both present in the cytosol, representing 21 % and 79 % of the total free *N*-oligosaccharides, respectively (Table 3). When protoplasts of *C. albicans* were treated with monensin, almost all the cytosolic *α*-mannosidase E-I was absent and the levels of M_7_B decreased to 3 % of the total content of cytosolic free *N*-oligosaccharides. This was accompanied by an increase in the M_8_B levels (Table 3). Similar results were obtained using protoplasts of *C. albicans* CAI4, SC5314 (data not shown) and *C. glabrata* (Table 3). When similar assays were performed using protoplasts of *S. cerevisiae, C. albicans kex2*Δ null mutant or *C. glabrata kex2*Δ null mutant, only M_8_B was detected in the cytosolic compartment (Table 3). Therefore the generation of M_7_B was associated with the presence of *α*1,2-mannosidase E-I in the cytosol.

## DISCUSSION

Core-*N*-glycan processing enzymes are of major importance to the growth and viability of fungi and play key roles in the biology of all eukaryotes. Our previous studies of *C. albicans* *α*-mannosidases showed that two activities could be identified and that about 85 % of soluble *α*1,2-mannosidase activity corresponds to enzyme E-II (Mora-Montes *et al.*, 2004). Here, cell disruption in the presence of pepstatin A generated membrane fractions with increased levels of *α*-mannosidase activity at the expense of a proportional decrease in the amount of soluble activity, which was enriched with *α*1,2-mannosidase E-I. These results suggest that pepstatin A inhibited the formation of *α*-mannosidase E-II. Hence, it is most likely that this enzyme corresponds to a soluble form of the membrane-bound *α*-mannosidase which is generated during sample preparation. Addition of pepstatin A did not affect the biochemical properties of purified *α*1,2-mannosidase E-I, which showed a molecular mass and *N*-mannan core processing similar to that previously described (Mora-Montes *et al.*, 2006). Accordingly, E-I can be classified in the glycosyl hydrolase family 47. The relationship of the 23 kDa polypeptide with the purified enzyme remains undetermined.

Density-gradient centrifugation assays indicated that *α*1,2-mannosidase activity of *C. albicans* protoplasts was associated with fractions with densities in the range reported for cytosolic, Golgi and ER compartments (Beaufay *et al.*, 1974; Chrispeels *et al.*, 1982; Chrispeels, 1983; Harris & Waters, 1996). This distribution contrasts with that exhibited by a similar homogenate from *S. cerevisiae* centrifuged under the same conditions, where *α*-mannosidase activity was separated into two peaks with densities in the range reported for Golgi and ER membranes.

Hexokinase I distribution confirmed that peak I was enriched with components from the cytosolic compartment. In agreement with the peak density range, a Golgi marker gave a strong signal in peak 2. This peak was also enriched with the Golgi form of CPY (Bryant & Stevens, 1998), which has a molecular mass (70 kDa) significantly higher than that of 61.1 kDa predicted for this enzyme (Mukhtar *et al.*, 1992). Peak 3 gave strong signals with ER but not Golgi and cytoplasmic markers, thus supporting the nature of these vesicles. The 68 kDa protein detected with the anti-CPY serum most likely corresponds to the ER form. Thus, the presence of hexokinase I and the absence of CPY and calnexin indicate that peak 1 did not contain vesicles or elements derived from the ER or Golgi. Because the marker for the Golgi compartment corresponds to a protein associated with the cytosolic face of the Golgi complex (Bloom & Brashear, 1989), it is possible that the barely detectable band in peak 1 represents trace quantities of the protein that is detached from the Golgi membranes. These results indicate that *α*1,2-mannosidase E-I is localized in the cytosolic cell fraction.

The anti-*α*1,2-mannosidase serum detected two polypeptides, of 52 kDa and 65 kDa, in Golgi and ER-associated vesicles, respectively. The molecular mass of 65 kDa is consistent with that predicted for the *C. albicans MNS1* gene product, and our results confirmed that this protein is Mns1, the ER membrane-associated *α*1,2-mannosidase (GenBank/EBI accession AY167027; Mora-Montes *et al.*, 2007). Its presence in the ER compartment probably results, as has been described for *S. cerevisiae* (Massaad & Herscovics, 2001), from a Rer1-dependent mechanism of localization. The enzyme E-I was not immunodetected in a *C. albicans mns1*Δ null mutant; thus, this gene encodes both *α*1,2-mannosidases. The members of glycosyl hydrolase family 47 that have so far been identified in the *C. albicans* genome (Arnaud *et al.*, 2005) are *MNS1* and orf19.834. The latter is homologous to *S. cerevisiae MNL1*, which encodes an ER mannosidase-like protein required for degradation of glycoproteins (Jakob *et al.*, 2001; Nakatsukasa *et al.*, 2001). However, these products have low values of identity (17.5 %) and similarity (28.5 %). *MNS1* and *AMS1*, the putative gene encoding the vacuolar *α*-mannosidase, showed identity and similarity of only 11.6 % and 19 %, respectively. These data reinforce the observation that both 65 kDa and 52 kDa polypeptides are encoded by *MNS1*.

Monensin blocked the transport of ER *α*1,2-mannosidase to Golgi and led to a depletion in the levels of *α*1,2-mannosidase E-I in the cytosolic compartment. These results suggest that the soluble *α*1,2-mannosidase is generated in the Golgi complex by proteolytic processing of the 65 kDa membrane-associated protein, and then is transported to the cytosolic compartment. Indeed, our results indicate that the 65 kDa membrane-bound enzyme was processed to *α*1,2-mannosidase E-I by Golgi Kex2 serine protease, which activates precursors of secreted proteins by an endoproteolytic cleavage at the C-terminus of dibasic motifs (Newport *et al.*, 2003). Moreover, inhibition of Kex2 by EGTA during the cell-breaking process did not change the *α*-mannosidase distribution in wild-type cells, suggesting that *α*1,2-mannosidase E-I is generated *in vivo* rather than *in vitro*. However, the membrane-bound *α*1,2-mannosidase from *S. cerevisiae* was not processed by Kex2. This is in agreement with previous reports indicating that ER membrane-bound *α*1,2-mannosidase is the unique isoform present *in vivo* in *S. cerevisiae* (Jelinek-Kelly & Herscovics, 1988; Herscovics, 1999a).

**Fig. 9. f9:**
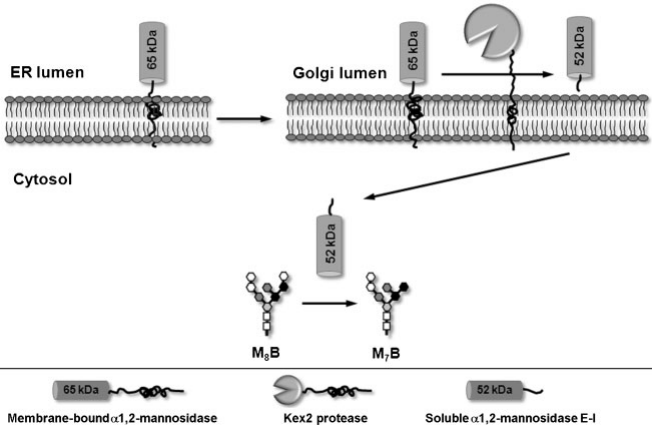
Model for the conversion of membrane-bound *α*1,2-mannosidase into soluble *α*1,2-mannosidase by Kex2. The ER membrane-bound *α*1,2-mannosidase is processed in the Golgi complex by Kex2 protease into soluble *α*1,2-mannosidase E-I. The soluble enzyme is then transported to the cytoplasmic compartment, where it is involved in the hydrolysis of soluble M_8_B oligosaccharide into M_7_B oligosaccharide.

The N-terminal sequence of both the purified *α*1,2-mannosidase E-I and the enzyme E-I proteolytically generated by Kex2 was located between amino acids 103 and 121 of the *MNS1* gene product. This predicts the generation of a soluble polypeptide of 463 amino acids with a molecular mass of 52.8 kDa, containing all the amino acids required for enzyme activity. These characteristics are fulfilled by *α*1,2-mannosidase E-I. Kex2 carries out the endoproteolysis at the C-terminus of dibasic amino acid motifs (Lys-Arg and Arg-Arg) (Fuller *et al.*, 1988). However, the N-terminal end of E-I is not preceded by a typical Kex2 recognition sequence. Instead, the sequences Ser^99^-Arg^100^ and Ala^101^-Arg^102^ are present. *In vitro* and *in vivo* studies have demonstrated that Kex2 can recognize both of these non-canonical sequences, but with lower affinity (Bevan *et al.*, 1998; Brenner & Fuller, 1992). This may explain why only around 45 % of the *α*-mannosidase activity was present as E-I. Hence, the results indicate that Kex2 recognizes an atypical cleavage site on the ER membrane-bound *α*1,2-mannosidase. The mechanism utilized to transport the *α*1,2-mannosidase E-I to the cytosolic compartment still remains to be determined. Fig. 9 shows a model for the processing of membrane-bound *α*1,2-mannosidase into enzyme E-I.

To gain further insights into the role of the cytosolic *α*1,2-mannosidase, we determined the levels of free cytosolic *N*-oligosaccharides in protoplasts treated and not treated with the ionophore monensin, which blocks glycosylation by affecting divalent cation transport into the Golgi. In agreement with previous reports (Chantret *et al.*, 2003), protoplasts of *S. cerevisiae* showed only the presence of M_8_B in the cytosolic preparations. It is believed that this *N*-oligosaccharide is generated by deglycosylation of glycoproteins during the ERAD (Chantret *et al.*, 2003). For protoplasts of *C. albicans*, M_8_B and M_7_B were detected in the cytosolic compartment, representing 21 % and 79 % of the total oligosaccharides, respectively. Protoplasts depleted of enzyme E-I had reduced amounts of M_7_B oligosaccharide and increased levels of M_8_B. These results suggest that enzyme E-I is required for the trimming of M_8_B into M_7_B. Similar results were obtained using protoplasts of *C. glabrata*, indicating that ER membrane-bound *α*1,2-mannosidase from this yeast can be processed to a cytosolic isoform by Kex2. Similar results have been reported in human cells, where the downregulation of cytosolic *α*-mannosidase activity directly influences the amount and the structure of the *N*-oligosaccharides present in the cytosol (Suzuki *et al.*, 2006).

The limited proteolysis of ER *α*1,2-mannosidase by Kex2 could represent a regulatory mechanism similar to that recently described for the human ER *α*1,2-mannosidase (Wu *et al.*, 2007), with implications for the regulation of ERAD, *N*-glycosylation and the degradation of cytosolic free *N*-oligosaccharides. To our knowledge this is the first report of a cytoplasmic *α*1,2-mannosidase in lower eukaryotes belonging to family 47 of glycosyl hydrolases.

## Figures and Tables

**Table 1. t1:** Effect of several protease inhibitors on the subcellular distribution of *α*-mannosidase activity in *C. albicans*

**Inhibitor**	Total *α*-mannosidase activity* [nmol MU min^−1^ (mg total protein)^−1^]	**Percentage of total activity**	
		**Supernatant**	**MMF†**
None	45±6	81±3	19±3
E64 (10 μM)	49±4	80±2	20±2
AEBSF (1 mM)	51±7	82±4	18±4
1,10-Phenanthroline (20 mM)	48±5	79±2	21±2
Pepstatin A (1 μM)	42±7	44±2	56±2
Inhibitor cocktail‡	49±8	78±3	22±3

*As measured with MU*α*Man as substrate. The values represent the means±sd of at least three independent cultures. †Mixed-membrane fraction. ‡Inhibitor of serine, cysteine and metalloproteases.

**Table 2. t2:** Purification of *α*1,2-mannosidase E-I from *C. albicans*

**Fraction**	**Total protein (mg)**	**Activity**		**Purification (*n*-fold)**	**Yield (%)**
		Specific*	**Total**		
High-speed supernatant	1010.0	0.05	50.5	1	100
Sepharose CL6B	125.8	0.8	96.6	16	191
DEAE Bio-Gel A	12.1	3.0	37.5	60	74
Sephadex G-25	10.8	3.4	36.7	68	73
DEAE Bio-Gel A	4.9	6.1	29.9	122	59
Con A-Sepharose 4B	1.9	10.9	20.7	218	41
Sephadex G-25	1.5	11.5	17.3	230	34

*Expressed as nmol MU min^−1^ (mg protein)^−1^.

**Table 3. t3:** Free *N*-oligosaccharides present in the cytosolic compartment

**Organism**	Cytosolic free *N*-oligosaccharides (μg)*	**M_8_B (%)†**	**M_7_B (%)†**
*C. albicans*:			
ATCC 26555	1.55±0.04‡	21	79
ATCC 26555+monensin‡	1.50±0.08	97	3
*kex2*Δ	1.49±0.06	100	nd
*kex2*Δ+monensin	1.53±0.09	100	nd
*C. glabrata*:			
ATCC 2001	1.38±0.09	19	81
ATCC 2001+monensin	1.32±0.08	98	2
*kex2*Δ	1.30±0.06	100	nd
*kex2*Δ+monensin	1.36±0.07	100	nd
*S. cerevisiae*	1.30±0.05	100	nd
*S. cerevisiae*+monensin	1.33±0.07	100	nd

*Per mg of cytosolic protein. The values represent the means±sd of at least three independent determinations. †Proportion of the total cytosolic free *N*-oligosaccharides. nd, Not detected. ‡Indicates protoplasts treated with monensin as described in Methods.

## Abbreviations

- 1-DMJ, 1-deoxymannojirimycin
- AEBSF, 4-(2-aminoethyl)benzenesulfonyl fluoride
- CPY, carboxypeptidase Y
- E64, *trans*-epoxysuccinyl-l-leucyl-amido(4-guanidino)butane
- ER, endoplasmic reticulum
- ERAD, endoplasmic-reticulum-associated degradation
- M_7_B, Man_7_GlcNAc_2_ isomer B
- M_8_B, Man_8_GlcNAc_2_ isomer B
- M_9_, Man_9_GlcNAc_2_
- MU, 4-methylumbelliferone
- MU*α*Man, 4-methylumbelliferyl-*α*-d-mannopyranoside
